# New species in *Dictyosporium*, new combinations in *Dictyocheirospora* and an updated backbone tree for Dictyosporiaceae

**DOI:** 10.3897/mycokeys.36.27051

**Published:** 2018-07-18

**Authors:** Jing Yang, Jian-Kui Liu, Kevin D. Hyde, E.B. Gareth Jones, Zuo-Yi Liu

**Affiliations:** 1 Guizhou Key Laboratory of Agricultural Biotechnology, Guizhou Academy of Agricultural Sciences, Guiyang 550006, Guizhou, China; 2 Center of Excellence in Fungal Research, Mae Fah Luang University, Chiang Rai 57100, Thailand; 3 Key Laboratory for Plant Diversity and Biogeography of East Asia, Kunming Institute of Botany, Chinese Academy of Science, Kunming 650201, Yunnan, China; 4 World Agroforestry Centre, East and Central Asia, 132 Lanhei Road, Kunming, 650201, Yunnan, China; 5 Department of Entomology and Plant Pathology, Faculty of Agriculture, Chiang Mai University, Huay Keaw Road, Suthep, Muang District, Chiang Mai 50200, Thailand

**Keywords:** 2 new taxa, asexual morph, Dothideomycetes, phylogeny, taxonomy

## Abstract

A survey of freshwater fungi on submerged wood in China and Thailand resulted in the collection of three species in *Dictyocheirospora* and four species in *Dictyosporium* including two new species in the latter genus. Morphological characters and phylogenetic analyses based on ITS, LSU and TEF1α sequence data support their placement in *Dictyocheirospora* and *Dictyosporium* (Dictyosporiaceae). An updated backbone tree is provided for the family Dictyosporiaceae. Descriptions and illustrations of the new taxa and re-collections are provided. Four new combinations are proposed for *Dictyocheirospora*.

## Introduction

The family Dictyosporiaceae was introduced by Boonmee et al. (2016) to accommodate mostly aquatic lignicolous species with cheiroid, digitate, palmate and/or dictyosporous conidia and their sexual morphs that form a monophyletic clade in the class Dothideomycetes.

*Dictyosporium*, the type genus of the family, has been reported worldwide from dead wood and plant litter in terrestrial and aquatic habitats (Hyde and Goh 1998, Ho et al. 2002, Pinnoi et al. 2006, Pinruan et al. 2007). Corda (1836) established the genus with *D. elegans* Corda as the type species. The holomorph genus is characterised by dark brown, subglobose superficial ascomata, bitunicate cylindrical asci and hyaline, fusiform uniseptate ascospores with or without a sheath; sporodochial colonies, micronematous to macronematous conidiophores and cheiroid, digitate complanate conidia with several parallel rows of cells. Goh et al. (1999) reviewed the genus accepting 22 species and the remaining 16 species were doubtful or excluded. Tsui et al. (2006) first considered that the genus is closely related to Massarinaceae (Pleosporales) based on phylogenetic analysis using SSU and LSU sequence data. Tanaka et al. (2015) and Boonmee et al. (2016) confirmed the phylogenetic placement of *Dictyosporium* in Dictyosporiaceae (Massarineae, Pleosporales). Recent comparisons of *Dictyosporium* species were provided by Whitton et al. (2012), Prasher and Verma (2015) and Silva et al. (2015) with up to 48 accepted species. Since Silva et al. (2015), *D. araucariae* S.S. Silva, R.F. Castañeda & Gusmão, *D. hydei* I.B. Prasher & R.K. Verma, *D. indicum* I.B. Prasher & R.K. Verma, *D. olivaceosporum* Kaz. Tanaka, K. Hiray., Boonmee & K.D. Hyde, *D. palmae* Abdel-Aziz, *D. pseudomusae* Kaz. Tanaka, G. Sato & K. Hiray., *D. sexualis* Boonmee & K.D. Hyde, *D. splendidum* Alves-Barb., Malosso & R.F. Castañeda and *D. wuyiense* Y. Zhang & G.Z. Zhao were newly introduced to the genus (Prasher and Verma 2015, Tanaka et al. 2015, Abdel-Aziz 2016, Boonmee et al. 2016, da Silva et al. 2016, Alves-Barbosa et al. 2017, Zhang et al. 2017) and nine species were re-assigned to *Dictyocheirospora*, *Jalapriya* and *Vikalpa* (Boonmee et al. 2016). Wijayawardene et al. (2017a) provided information on the availability of cultures and references to accessible sequence data.

*Dictyocheirospora* was introduced by Boonmee et al. (2016) with *Di. rotunda* D’souza, Bhat & K.D. Hyde as the type species. *Dictyocheirospora* is morphologically similar to *Dictyosporium* except in having cheiroid, non-complanate or cylindrical conidia, mostly with conidial arms closely gathered together at the apex. Ten species are accepted in the genus including four species transferred from *Dictyosporium* (Boonmee et al. 2016, Wang et al. 2016, Hyde et al. 2017, Li et al. 2017).

During a survey of freshwater fungi on submerged wood along a north/south gradient in the Asian/Australasian region (Hyde et al. 2016), two new freshwater species and five previously described species were collected and identified based on phylogenetic analyses and morphological characters. We therefore introduce *Dictyosporium tubulatum* and *Dictyosporium tratense* as new species, with an illustrated account and phylogenetic evidence for the new taxa. An updated backbone tree based on the combined ITS, LSU and TEF1α sequence data is provided for Dictyosporiaceae. Four new combinations are proposed in *Dictyocheirospora*.

## Materials and methods

### Collection and examination of specimens

Specimens of submerged, decaying wood were collected from streams in Chiang Rai, Prachuap Khiri Khan, Phang Nga and Trat Provinces, Thailand, in December 2014, 2015, April 2016 and Guizhou Province, China, in October 2016. Specimens were brought to the laboratory in plastic bags and incubated in plastic boxes lined with moistened tissue paper at room temperature for one week. Morphological observations were made using a Motic SMZ 168 Series dissecting microscope for fungal structures on natural substrate. The fungal structures were collected using a syringe needle and transferred to a small drop of distilled water on a clean slide and covered with a cover glass. The fungi were examined using a Nikon ECLIPSE 80i compound microscope and photographed with a Canon 550D, 600D or 70D digital camera fitted to the microscope. Measurements were made with the TAROSOFT (R) IMAGE FRAME WORK programme and images used for figures were processed with ADOBE PHOTOSHOP CS6 software. Single spore isolations were made on to potato dextrose agar (PDA) or water agar (WA) and later transferred on to malt extract agar (MEA) or PDA following the method of Chomnunti et al. (2014). Specimens (dry wood with fungal material) are deposited in the herbarium of Mae Fah Luang University (MFLU), Chiang Rai, Thailand and Kunming Institute of Botany, Academia Sinica (HKAS), China. Axenic cultures are deposited in Mae Fah Luang University Culture Collection (MFLUCC). Facesoffungi and Index Fungorum numbers are registered as outlined in Jayasiri et al. (2015) and Index Fungorum (2018).

### DNA extraction, PCR amplification and sequencing

Isolates were grown on PDA and/or MEA medium at 25 °C for one month. Fungal mycelium was scraped off and transferred to a 1.5-ml microcentrifuge tube using a sterilised lancet for genomic DNA extraction. Ezup Column Fungi Genomic DNA Purification Kit (Sangon Biotech, China) was used to extract DNA following the manufacturer’s instructions. ITS, LSU and TEF1α gene regions were amplified using the primer pairs ITS5 or ITS1 with ITS4 (Vilgalys and Hester 1990), LROR with LR5 or LR7 (White et al. 1990) and EF1-983F with EF1-2218R (Rehner 2001). The amplifications were performed in a 25 μl reaction volume containing 9.5 μl ddH_2_O, 12.5 μl 2 × Taq PCR Master Mix with blue dye (Sangon Biotech, China), 1 μl of DNA template and 1 μl of each primer (10 μM). The amplification condition for ITS, LSU and TEF1α consisted of initial denaturation at 94 °C for 3 min; followed by 40 cycles of 45 s at 94 °C, 50 s at 56 °C and 1 min at 72 °C and a final extension period of 10 min at 72 °C. Purification and sequencing of PCR products were carried out using the above-mentioned PCR primers at Sangon Biotech (Shanghai) Co. Ltd. in China.

### Phylogenetic analyses

The taxa included in the phylogenetic analyses were selected and obtained from previous studies and GenBank (Boonmee et al. 2016, Wang et al. 2016, Li et al. 2017). Three gene regions (ITS, LSU and TEF1α) were used for the combined sequence data analyses. SEQMAN v. 7.0.0 (DNASTAR, Madison, WI) was used to assemble consensus sequences. The sequences were aligned using the online multiple alignment programme MAFFT v.7 (http://mafft.cbrc.jp/alignment/server/) (Katoh and Standley 2013). The alignments were checked visually and improved manually where necessary.

Phylogenetic analysis of the sequence data consisted of maximum likelihood (ML) using RAxML-HPC v.8 (Stamatakis 2006, Stamatakis et al. 2008) on the XSEDE Teragrid of the CIPRES science Gateway (https://www.phylo.org) (Miller et al. 2010) with rapid bootstrap analysis, followed by 1000 bootstrap replicates. The final tree was selected amongst suboptimal trees from each run by comparing likelihood scores under the GTRGAMMA substitution model.

Maximum parsimony (MP) analyses were performed with PAUP v. 4.0b10 (Swofford 2003) using the heuristic search option with 1000 random taxa addition and tree bisection and reconnection (TBR) as the branch swapping algorithm. All characters were unordered and of equal weight and gaps were treated as missing data. Maxtrees were unlimited, branches of zero length were collapsed and all multiple, equally parsimonious trees were saved. Clade stability was assessed using a bootstrap (BT) analysis with 1000 replicates, each with 10 replicates of random stepwise addition of taxa (Hillis and Bull 1993).

The programme MRMODELTEST2 v. 2.3 (Nylander 2008) was used to infer the appropriate substitution model that would best fit the model of DNA evolution for the combined datasets for Bayesian inference analysis with GTR+G+I substitution model selected. Posterior probabilities (PP) (Rannala and Yang 1996, Zhaxybayeva and Gogarten 2002) were determined by Markov Chain Monte Carlo sampling (MCMC) in MRBAYES v. 3.0b4 (Huelsenbeck and Ronquist 2001). Six simultaneous Markov chains were run for 1 million generations, with trees sampled every 100 generations (resulting in 10000 trees). The first 2000 trees, representing the burn-in phase of the analyses were discarded and the remaining 8000 trees were used for calculating posterior probabilities (PP) in the majority rule consensus tree (Larget and Simon 1999).

The resulting trees were printed with FIGTREE v. 1.4.0 (http://tree.bio.ed.ac.uk/software/figtree/) and the layout was created in MICROSOFT POWERPOINT for Mac v. 15.19.1. The alignment of phylogenetic analyses and resultant tree were deposited in TreeBASE (www.treebase.org, submission number 22802). Sequences generated in this study were submitted to GenBank (Table 1).

**Table 1.** Isolates and sequences used in this study (newly generated sequences are indicated in bold, ex-type strains are indicated with ^T^ after strain number).

Species	Source	GenBank accession number		
		ITS	LSU	TEF1α
*Aquadictyospora lignicola*	MFLUCC 17-1318^T^	MF948621	MF948629	MF953164
*Aquaticheirospora lignicola*	HKUCC 10304^T^	AY864770	AY736378	–
*Cheirosporium triseriale*	HMAS 180703^T^	EU413953	EU413954	–
*Dendryphiella eucalyptorum*	CBS 137987^T^	KJ869139	KJ869196	–
*Dendryphiella fasciculata*	MFLUCC 17-1074^T^	MF399213	MF399214	–
*Dendryphiella paravinosa*	CBS 141286^T^	KX228257	KX228309	–
*Dictyocheirospora aquatica*	KUMCC 15-0305^T^	KY320508	KY320513	–
*Dictyocheirospora bannica*	KH 332^T^	LC014543	AB807513	AB808489
***Dictyocheirospora bannica***	**MFLUCC 16-0874**	**MH381765**	**MH381774**	–
*Dictyocheirospora garethjonesii*	MFLUCC 16-0909^T^	KY320509	KY320514	–
*Dictyocheirospora garethjonesii*	DLUCC 0848	MF948623	MF948631	MF953166
*Dictyocheirospora gigantica*	BCC 11346	DQ018095	–	–
*Dictyocheirospora heptaspora*	CBS 396.59	DQ018090	–	–
***Dictyocheirospora indica***	**MFLUCC 15-0056**	**MH381763**	**MH381772**	**MH388817**
*Dictyocheirospora pseudomusae*	yone 234^T^	LC014550	AB807520	AB808496
*Dictyocheirospora rotunda*	MFLUCC 14-02^9^3T	KU179099	KU179100	–
***Dictyocheirospora rotunda***	**MFLUCC 17-0222**	**MH381764**	**MH381773**	**MH388818**
*Dictyocheirospora rotunda*	MFLUCC 17-1313	MF948625	MF948633	MF953168
*Dictyocheirospora subramanianii*	BCC 3503	DQ018094	–	–
*Dictyocheirospora vinaya*	MFLUCC 14-0294^T^	KU179102	KU179103	–
*Dictyosporium alatum*	ATCC 34953^T^	NR_077171	DQ018101	–
*Dictyosporium aquaticum*	MF 1318^T^	KM610236	–	–
*Dictyosporium bulbosum*	yone 221	LC014544	AB807511	AB808487
*Dictyosporium digitatum*	KH 401	LC014545	AB807515	AB808491
*Dictyosporium digitatum*	yone 280	LC014547	AB807512	AB808488
*Dictyosporium elegans*	NBRC 32502^T^	DQ018087	DQ018100	–
*Dictyosporium hughesii*	KT 1847	LC014548	AB807517	AB808493
*Dictyosporium meiosporum*	MFLUCC 10-0131^T^	KP710944	KP710945	–
*Dictyosporium nigroapice*	BCC 3555	DQ018085	–	–
***Dictyosporium nigroapice***	**MFLUCC 17-2053**	**MH381768**	**MH381777**	**MH388821**
*Dictyosporium olivaceosporum*	KH 375^T^	LC014542	AB807514	AB808490
*Dictyosporium sexualis*	MFLUCC 10-0127^T^	KU179105	KU179106	–
***Dictyosporium* sp.**	**MFLUCC 15-0629**	**MH381766**	**MH381775**	**MH388819**
*Dictyosporium stellatum*	CCFC 241241^T^	NR_154608	JF951177	–
*Dictyosporium strelitziae*	CBS 123359^T^	NR_156216	FJ839653	–
*Dictyosporium tetrasporum*	KT 2865	LC014551	AB807519	AB808495
*Dictyosporium thailandicum*	MFLUCC 13-0773^T^	KP716706	KP716707	–
***Dictyosporium tratense***	**MFLUCC 17-2052^T^**	**MH381767**	**MH381776**	**MH388820**
***Dictyosporium tubulatum***	**MFLUCC 15-0631^T^**	**MH381769**	**MH381778**	**MH388822**
***Dictyosporium tubulatum***	**MFLUCC 17-2056**	**MH381770**	**MH381779**	–
*Dictyosporium wuyiense*	CGMCC 3.18703^T^	KY072977	–	–
*Dictyosporium zhejiangense*	MW-2009a^T^	FJ456893	–	–
*Digitodesmium bambusicola*	CBS 110279^T^	DQ018091	DQ018103	–
*Gregarithecium curvisporum*	KT 922^T^	AB809644	AB807547	–
*Jalapriya inflata*	NTOU 3855	JQ267362	JQ267363	–
*Jalapriya pulchra*	MFLUCC 15-0348^T^	KU179108	KU179109	–
*Jalapriya pulchra*	MFLUCC 17-1683	MF948628	MF948636	MF953171
*Jalapriya toruloides*	CBS 209.65	DQ018093	DQ018104	–
*Periconia igniaria*	CBS 379.86	LC014585	AB807566	AB808542
*Periconia igniaria*	CBS 845.96	LC014586	AB807567	AB808543
*Pseudocoleophoma calamagrostidis*	KT 3284^T^	LC014592	LC014609	LC014614
*Pseudocoleophoma polygonicola*	KT 731^T^	AB809634	AB807546	AB808522
*Pseudocoleophoma typhicola*	MFLUCC 16-0123^T^	KX576655	KX576656	–
*Pseudodictyosporium elegans*	CBS 688.93^T^	DQ018099	DQ018106	–
*Pseudodictyosporium indicum*	CBS 471.95	DQ018097	–	–
*Pseudodictyosporium thailandica*	MFLUCC 16-0029^T^	KX259520	KX259522	KX259526
*Pseudodictyosporium wauense*	NBRC 30078	DQ018098	DQ018105	–
*Pseudodictyosporium wauense*	DLUCC 0801	MF948622	MF948630	MF953165
*Vikalpa australiensis*	HKUCC 8797^T^	DQ018092	–	–

### Phylogenetic results

**Figure 1.**
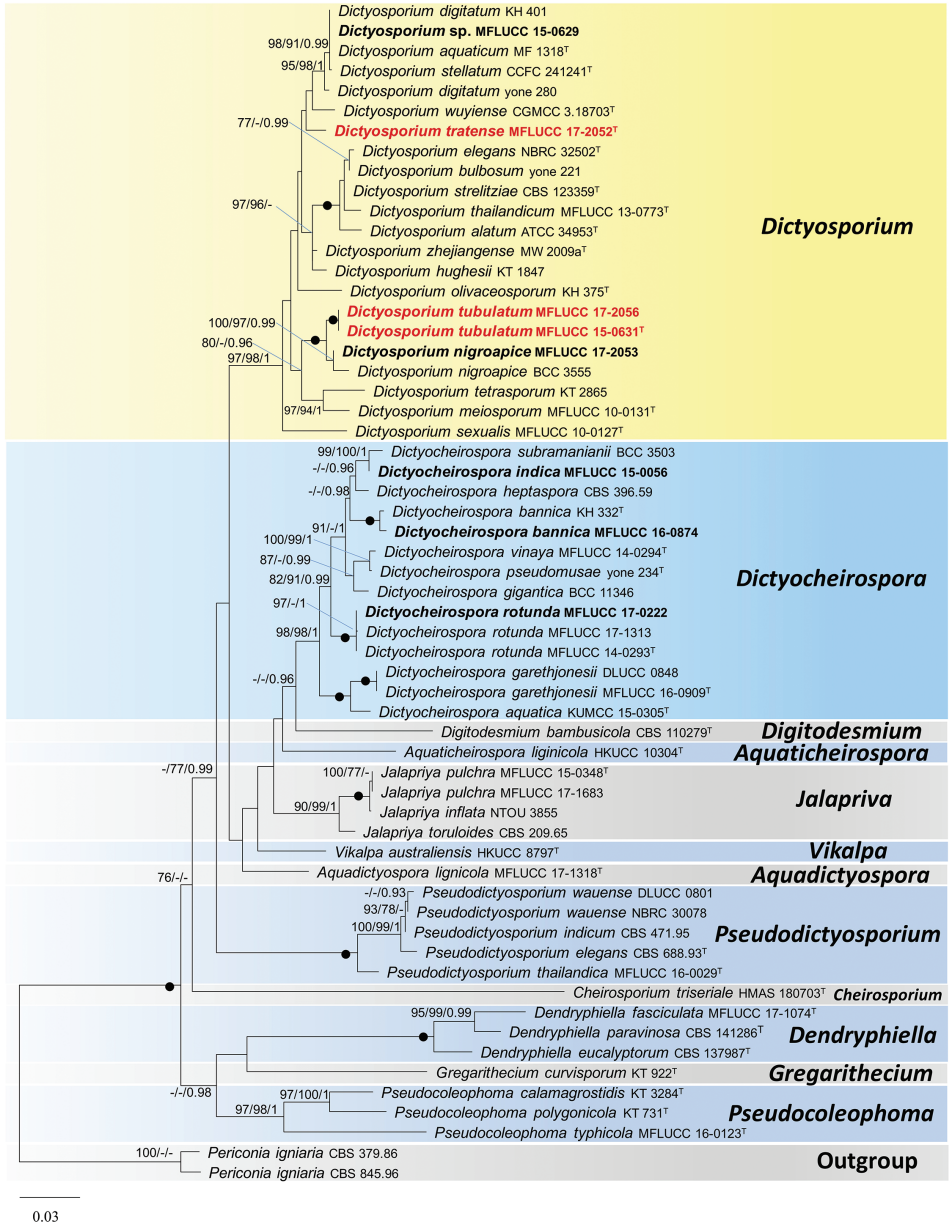
Maximum likelihood majority rule consensus tree for the analysed Dictyosporiaceae isolates based on a dataset of combined ITS, LSU and TEF1α sequence data. Bootstrap support values for maximum likelihood (ML) and maximum parsimony (MP) greater than 75% and Bayesian posterior probabilities greater than 0.95 are indicated above the nodes as MLBS/MPBS/PP. The scale bar represents the expected number of changes per site. The tree is rooted with *Periconia igniaria* (CBS 379.86, CBS 845.96). The strain numbers are noted after the species names with ex-type strains indicated with ^T^. The new collections are in bold with new taxa in red. Branches with 100% ML BS, 100% MP BS and 1.0 PP are shown as black nodes. Genera are indicated as coloured blocks.

The analysed dataset consisted of combined ITS (557 bp), LSU (803 bp) and TEF1α (918 bp) sequence data (a total of 2278 characters including gaps) for 59 taxa in Dictyosporiaceae with *Periconia igniaria* E.W. Mason & M.B. Ellis (CBS 379.86, CBS 845.96) as the outgroup taxon. The best scoring RAxML tree is shown in Figure 1.

Phylogenetic analyses indicated the placement of three isolates (MFLUCC 15-0056, MFLLUCC 16-0874 and MFLUCC 17-0222) within the genus *Dictyocheirospora*. Five isolates (MFLUCC 15-0629, MFLUCC 17-2052, MFLUCC 17-2056, MFLUCC 15-0631 and MFLUCC 17-2053) nested in *Dictyosporium*. Phylogenetic results showed that *Dictyocheirospora indica* (MFLUCC 15-0056) clustered with *Dictyocheirospora subramanianii* (B. Sutton) D'souza, Boonmee & K.D. Hyde (BCC 3503) with good support. *Dictyocheirospora bannica* (MFLUCC 16-0874) was placed as sister taxon to the ex-type strain *Dictyocheirospora bannica* (KH 332). *Dictyocheirospora rotunda* (MFLUCC 17-0222) grouped together with *Dictyocheirospora rotunda* (MFLUCC 17-1313) and the ex-type strain *Dictyocheirospora rotunda* (MFLUCC 14-0293) with strong support. The strain *Dictyosporium* sp. (MFLUCC 15-0629) clustered as sister taxon to *Dictyosporium digitatum* J.L. Chen, C.H. Hwang & Tzean (KH 401), *Dictyosporium aquaticum* Abdel-Aziz (MF 1318) and *Dictyosporium stellatum* G.P. White & Seifert (CCFC 241241). The new taxon *Dictyosporium tratense* (MFLUCC 17-2052) formed a single clade within *Dictyosporium* which is distinct from other species in the genus. The new collection *Dictyosporium nigroapice* (MFLUCC 17-2053) was placed as sister taxon to a previous isolate *Dictyosporium nigroapice* (BCC 3555). Two isolates of the new taxon *Dictyosporium tubulatum* (MFLUCC 15-0631 and MFLUCC 17-2056) nested in *Dictyosporium* as sister clade to *Dictyosporium nigroapice* (MFLUCC 17-2053 and BCC 3555).

## Taxonomy

### *Dictyocheirospora* species

#### Dictyocheirospora bannica

Taxon classificationFungiPleosporalesDictyosporiaceae

Kaz. Tanaka, K. Hiray., Boonmee & K.D. Hyde, Fungal Diversity 80: 467 (2016)

Figure 2

##### Material examined.

THAILAND. Phang Nga Province, Bann Tom Thong Khang, on decaying wood submerged in a freshwater stream, 17 Dec 2015, J. Yang, Site 7-70-1 (MFLU 18-1040, HKAS 102131), living culture MFLUCC 16-0874 (Additional SSU sequence GenBank MH381759).

##### Notes.

The phylogenetic result showed the strain MFLUCC 16-0874 clustered with the ex-type (KH 332) of *Dictyocheirospora bannica*. The morphological examination of this collection matched well with the holotype of *Dictyocheirospora bannica* (Boonmee et al. 2016). *Dictyocheirospora bannica* was previously collected in Japan, while this is a new record for Thailand.

**Figure 2.**
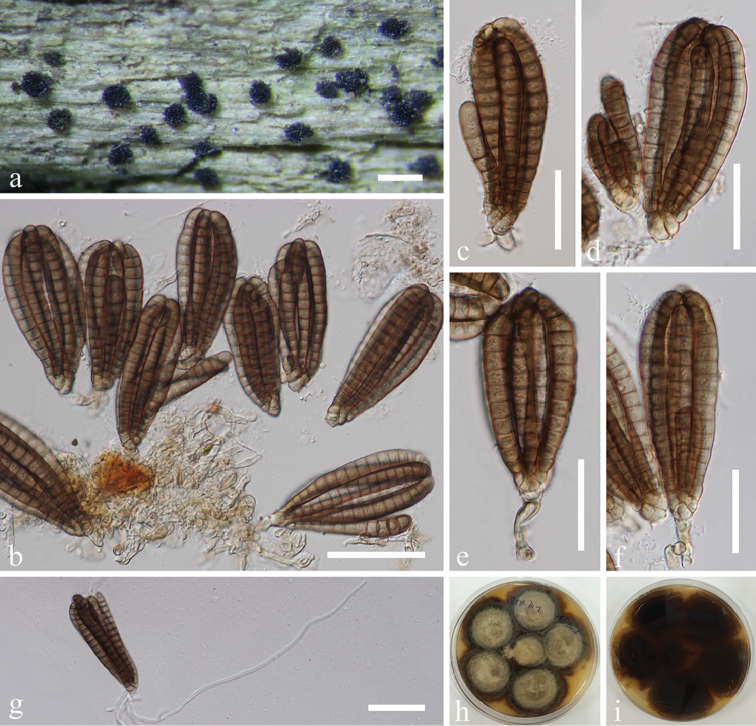
*Dictyocheirospora bannica* (MFLU 18-1040) **a** Colonies on submerged wood **b** Conidia and conidiophores **c–f** Conidia **g** Germinated conidium **h, i** Culture, h from above, i from reverse. Scale bars: **a** = 200 μm, **b, g** = 50 μm, **c–f** = 30 μm.

#### Dictyocheirospora hydei

Taxon classificationFungiPleosporalesDictyosporiaceae

(I.B. Prasher & R.K. Verma) J. Yang & K.D. Hyde comb. nov.

##### Basionym.

*Dictyosporium hydei* I.B. Prasher & R.K. Verma, Phytotaxa 204 (3): 196 (2015).

##### Holotype.

INDIA. Himachal Pradesh, Bilaspur, on bark of *Tecoma stans*, 17 September 2013, I.B. Prasher and R.K. Verma (PAN 30364).

##### Notes.

Considering the latest generic concept of *Dictyocheirospora* and *Dictyosporium*, we suggest that *Dictyosporium hydei* should be referred to *Dictyocheirospora* with the key character of non-complanate or cylindrical conidia with conidial arms closely gathered together at the apex. We have not examined the holotype of *Dictyocheirospora hydei*. The details provided by Prasher and Verma (2015) are adequate being illustrative and descriptive.

#### Dictyocheirospora indica

Taxon classificationFungiPleosporalesDictyosporiaceae

(I.B. Prasher & R.K. Verma) J. Yang & K.D. Hyde comb. nov.

Figure 3

##### Basionym.

*Dictyosporium indicum* I.B. Prasher & R.K. Verma, Phytotaxa 204 (3): 194 (2015).

##### Holotype.

INDIA. Himachal Pradesh, Mandi, on petiole of *Phoenix rupicola*, 19 November 2012, I.B. Prasher and R.K. Verma (PAN 30313).

##### Material examined.

THAILAND. Chiang Rai, stream flowing in Tham Luang Nang Non Cave, on decaying submerged wood, 25 November 2014, J. Yang, YJ-3 (MFLU 15-1169 **reference specimen designated here**, HKAS 102135), living culture MFLUCC 15-0056 (Additional SSU sequence GenBank MH381757).

##### Notes.

Collection MFLU 15-1169 was identified as *Dictyocheirospora indica* (*Dictyosporium indicum*) based on morphological examination. Phylogenetic analyses indicated the placement of this taxon within *Dictyocheirospora* and sister to *Di. subramanianii* (BCC 3503). *Dictyocheirospora subramanianii* differs from *Di. indica* in lacking appendages. *Dictyocheirospora indica* resembles *Di. musae* in having non-complanate, cylindrical conidia with globose to subglobose appendages. However, conidial appendages of *Di. indica* are attached at the subapical cells, while appendages of *Di. musae* are attached at the central cells of the outer cell-row. The conidial size of *Di. indica* (33–48 × 13–18 µm) is smaller than that of *Di. musae* (45–65 × 20–27 µm) (Photita et al. 2002, Prasher and Verma 2015). In this study, sequence data of our collection *Dictyocheirospora indica* (MFLUCC 15-0056) was generated and, as there is no sequence data available for the previous collection (*Dictyosporium indicum*), we therefore designated our collection as the reference specimen (*sensu* Ariyawansa et al. 2014) for *Dictyocheirospora indica*.

**Figure 3.**
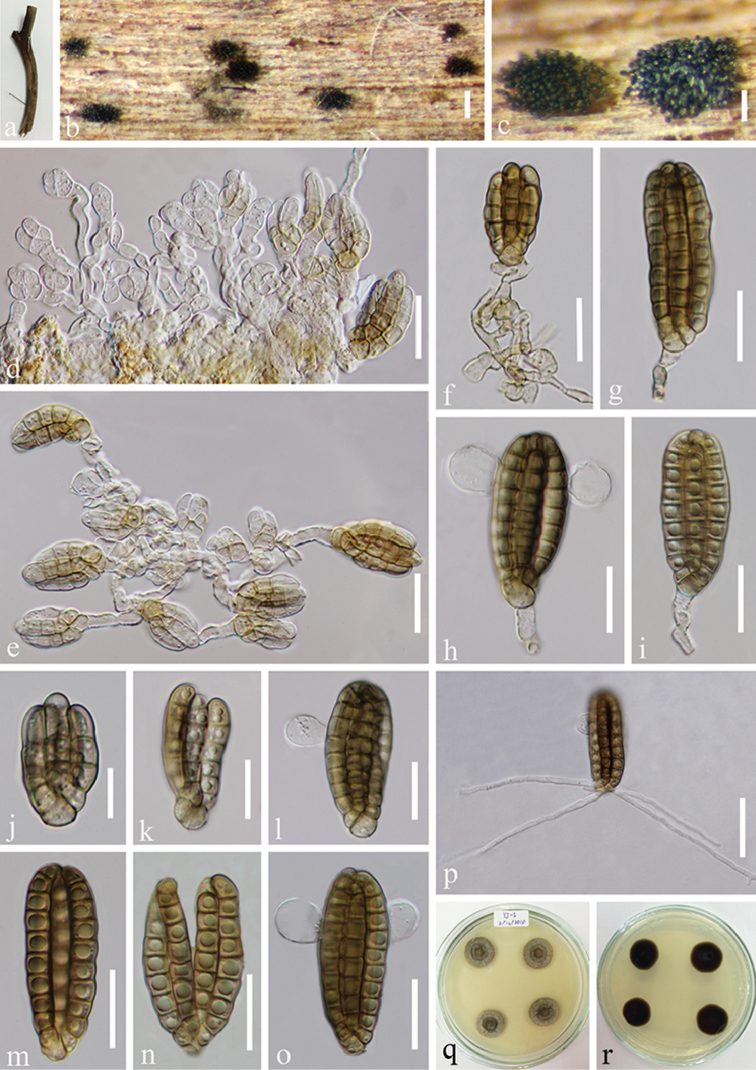
*Dictyocheirospora indica* (MFLU 15-1169, reference specimen). **a** Substrate **b, c** Colonies on woody substrate **d, e** Conidial formation **f–i** Conidia with partial conidiophores **j–o** Conidia **p** Germinated conidium **q–r** Culture, q from above, r from reverse. Scale bars: **b** = 200 μm, **c** = 100 μm, **d–i, l–o** = 20 μm, **j** = 10 μm, **k** = 15 μm, **p** = 30 μm.

#### Dictyocheirospora musae

Taxon classificationFungiPleosporalesDictyosporiaceae

(Photita) J. Yang, K.D. Hyde & Z.Y. Liu comb. nov.

##### Basionym.

*Dictyosporium musae* Photita, Mycotaxon 82: 416 (2002)

##### Holotype.

THAILAND. Mae Hong Son Province, Sob Mei, Huay Thicha Village, on decaying petioles of *Musa acuminata*, 23 November 2000, W. Photita (PDD 74135).

**Notes.** *Dictyocheirospora musae* is morphologically similar to *Di. hydei* in having non-complanate, cylindrical conidia with globose to subglobose appendages. However, *Dictyocheirospora musae* differs in having appendages in the middle cells while *Di. hydei* has appendages on the basal cells (Photita et al. 2002, Prasher and Verma 2015).

#### Dictyocheirospora rotunda

Taxon classificationFungiPleosporalesDictyosporiaceae

D’souza, Bhat & K.D. Hyde, Fungal Diversity 80: 465 (2016)

Figure 4

##### Material examined.

CHINA. Guizhou Province, Anshun city, Gaodang village, 26°4.267'N, 105°41.883'E, on decaying wood submerged in Suoluo river, 19 October 2016, J. Yang, GD 2-3 (MFLU 18-1041, HKAS 102132), living culture MFLUCC 17-0222 (Additional SSU sequence GenBank MH381758).

##### Notes.

This species is known in China and Thailand from freshwater habitats (Boonmee et al. 2016, Wang et al. 2016).

**Figure 4.**
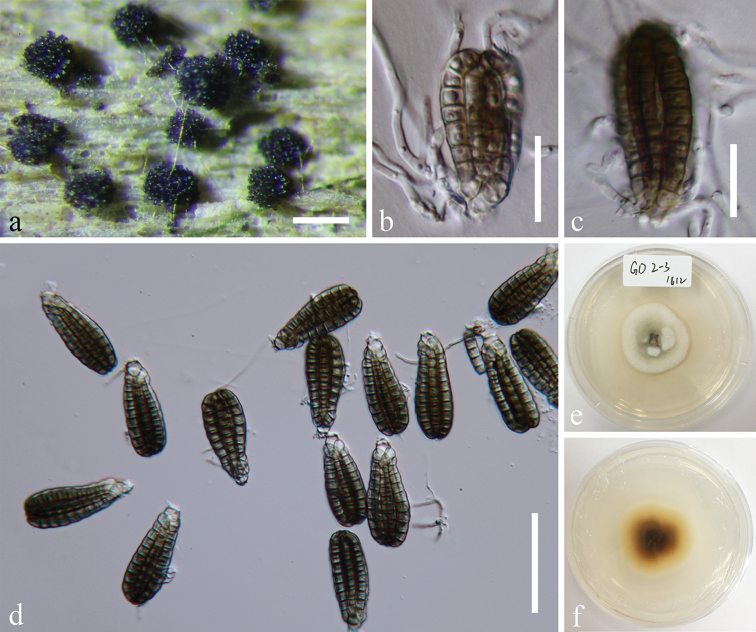
*Dictyocheirospora rotunda* (MFLU 18-1041). **a** Colonies on submerged wood **b, c** Germinated conidia **d** Conidia **e, f** Culture, e from above, f from reverse. Scale bars: **a** = 200 μm, **b, c** = 20 μm, **d** = 50 μm.

#### Dictyocheirospora tetraploides

Taxon classificationFungiPleosporalesDictyosporiaceae

(L. Cai & K.D. Hyde) J. Yang & K.D. Hyde comb. nov.

##### Basionym.

*Dictyosporium tetraploides* L. Cai & K.D. Hyde, Sydowia 55 (2): 132 (2003)

##### Holotype.

CHINA. Yunnan, Xishuangbanna, Menglun, a small stream, on submerged wood, 21 June 2002, L. Cai (HKUM 17146).

##### Notes.

*Dictyocheirospora tetraploides* is morphologically similar to *Di. musae* in conidial shape, size, colour and appendages. However, conidia of *Di. tetraploides* have 5-rowed cells, while those of *Di. musae* are 7-rowed cells (Photita et al. 2002, Cai et al. 2003).

### *Dictyosporium* species

#### Dictyosporium tubulatum

Taxon classificationFungiPleosporalesDictyosporiaceae

J. Yang, K.D. Hyde & Z.Y. Liu sp. nov.

Figure 5

##### Etymology.

Referring to the tubular conidial appendages.

##### Description.

*Saprobic* on decaying plant substrates. **Asexual morph**: *Colonies* punctiform, sporodochial, scattered, dark brown to black, glistening. *Mycelium* mostly immersed, composed of smooth, septate, branched, hyaline to pale brown hyphae. *Conidiophores* micronematous, mononematous, septate, cylindrical, hyaline to pale brown, smooth-walled, 6.5–15 × 3.5–6 μm, sometimes reduced to conidiogenous cells. *Conidiogenous cells* monoblastic, integrated, terminal, determinate, hyaline to pale brown. *Conidia* acrogenous, solitary, cheiroid, smooth-walled, complanate, yellowish-brown to medium brown, mostly consisting of four arms closely compact with side arms lower than middle arms, rarely with five arms, 5–7-euseptate in each arm, guttulate, (22–)29–35(–38) × (14–)17–19(–22) μm (x¯ = 32.5 × 18 μm, n = 40), with hyaline, tubular, elongated appendages which are 19–24 × 3.5–7 μm and mostly attached at the apical part of two outer arms. **Sexual morph**: Undetermined.

##### Cultural characteristics.

Conidia germinating on PDA within 24 h and germ tubes produced from the basal cell. Colonies on MEA reaching 5–10 mm diam. in a week at 25 °C, in natural light, circular, with fluffy, dense, white mycelium on the surface with entire margin; in reverse yellow in the middle and white at the margin.

##### Material examined.

THAILAND. Prachuap Khiri Khan Province, near 12°30.19'N, 99°31.35'E, on decaying wood submerged in a freshwater stream, 25 December 2014, J. van Strien, Site 5-11-1 (MFLU 15-1166 **holotype**, HKAS 102136 **isotype**), ex-type living culture MFLUCC 15-0631; *ibid.* Trat Province, Amphoe Ko Chang, 12°08'N, 102°38'E, on decaying wood submerged in a freshwater stream, 27 April 2017, Y.Z. Lu, YJT 22-2 (MFLU 18-1044, HKAS 102137 **paratype**), living culture MFLUCC 17-2056.

##### Notes.

Phylogenetic analyses showed that *Dictyosporium tubulatum* nested in *Dictyosporium* and sister to *D. nigroapice*. *Dictyosporium tubulatum* morphologically resembles *D. alatum* Emden, *D. canisporum* L. Cai & K.D. Hyde and *D. thailandicum* D’ souza, D.J. Bhat & K.D. Hyde in conidial ontogeny and conidial shape, colour and appendages. *Dictyosporium tubulatum* differs from the three species in the number of conidial cell rows. There are mostly four conidial columns in *D. tubulatum* while mostly five columns in the others. *Dictyosporium tubulatum* has smaller conidia (25–38 × 14–22 μm) than those in *D. canisporum* (32.5–47.5 × 20–25 μm) but has similar conidial size with *D. alatum* (26–32 × 15–24 μm) and *D. thailandicum* (15.4–34.5 × 14.5–20.6 μm) (Cai et al. 2003, Liu et al. 2015). Based on the molecular phylogeny, *D. tubulatum* is distinct from *D. thailandicum* and *D. alatum*. Unfortunately, molecular data are unavailable for *D. canisporum*.

**Figure 5.**
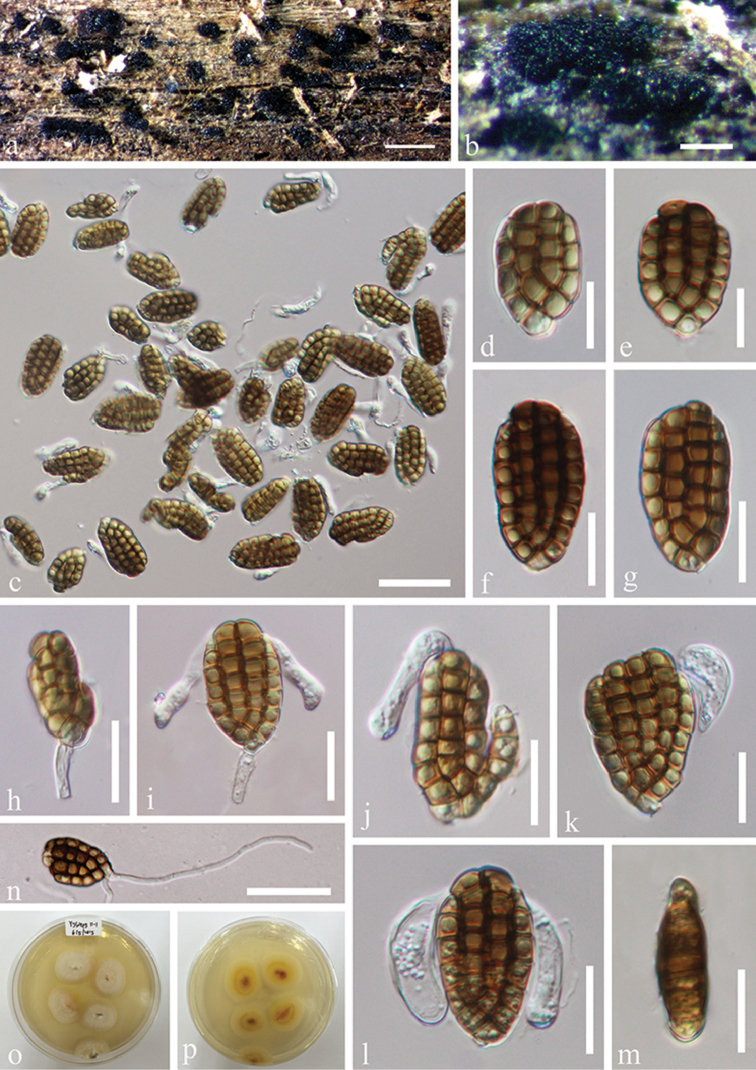
*Dictyosporium tubulatum* (MFLU 15-1166, holotype). **a, b** Colonies on woody substrate **c** Squash mount of a sporodochium **d–g** Conidia **h–i** Conidia with conidiophores **j–l** Conidia with appendages **m** lateral view of a conidium **n** Germinated conidium **o, p** Culture, o from above **p** from reverse. Scale bars: **a** = 1000 μm, **b** = 200 μm, **c, n** = 30 μm, **d, e** = 10 μm, **f–m** = 15 μm.

#### Dictyosporium tratense

Taxon classificationFungiPleosporalesDictyosporiaceae

J. Yang & K.D. Hyde sp. nov.

Figure 6

##### Etymology.

Referring to the collecting site in Trat province, Thailand.

##### Description.

*Saprobic>* on decaying plant substrates. **Asexual morph**: *Colonies* punctiform, sporodochial, scattered, black, glistening. *Mycelium* mostly immersed, composed of smooth, septate, branched, hyaline to pale brown hyphae. *Conidiophores* micronematous, mononematous, septate, cylindrical, hyaline to pale brown, smooth-walled, sometimes reduced to conidiogenous cells. *Conidiogenous cells* monoblastic, integrated, terminal, determinate, hyaline to pale brown. *Conidia* (40–)43–54(–57) × (20–)23–32(–36) μm (x¯ = 49.5 × 26 μm, n = 40), acrogenous, solitary, cheiroid, smooth-walled, complanate, yellowish-brown to light brown, consisting of 39–68 cells arranged in 4–6 (mostly 5) closely compact columns, 9–11-euseptate in each column, guttulate; the inner columns nested within the outer columns, the outer columns derived from the basal cell of the conidium; the intermediate columns are derived from the first or second cell of the outer columns; the inner columns derived from the first or second cell of the intermediate columns; usually with 2–3 central columns longest and of equal length, 2–3 peripheral columns shorter and of equal length; sometimes with hyaline globose appendages at the apical cells of outer columns with hyaline cloud-shaped mucilaginous sheath. **Sexual morph**: Undetermined.

##### Cultural characteristics.

Conidia germinating on PDA within 24 h and germ tubes produced from basal cell. Colonies on MEA reaching 5–10 mm diam. in a week at 25 °C, in natural light, circular, with fluffy, dense, pale yellow mycelium in the middle and sparse mycelium in the outer ring on the surface with irregular margin; in reverse, dark yellow to brown in the middle and pale yellow at the margin.

##### Material examined.

THAILAND. Trat Province, Amphoe Ko Chang, 12°08'N, 102°38'E, on decaying wood submerged in a freshwater stream, 27 April 2017, Y.Z. Lu, YJT 6-2 (MFLU 18-1042 **holotype**, HKAS 102133 **isotype**), ex-type living culture MFLUCC 17-2052 (Additional SSU sequence GenBank MH381761).

##### Notes.

Phylogenetic analyses indicated *Dictyosporium tratense* nested within *Dictyosporium* and close to *D. wuyiense*. It is distinguished from the other species in the genus in having a mucilaginous sheath. Morphologically, *D. tratense* is most comparable to *D. elegans* in conidial colour and shape, but conidia of the new taxon (40-57 × 20-36 μm) are smaller than those of *D. elegans* (40-80 × 24-36 μm) (Goh et al. 1999).

**Figure 6.**
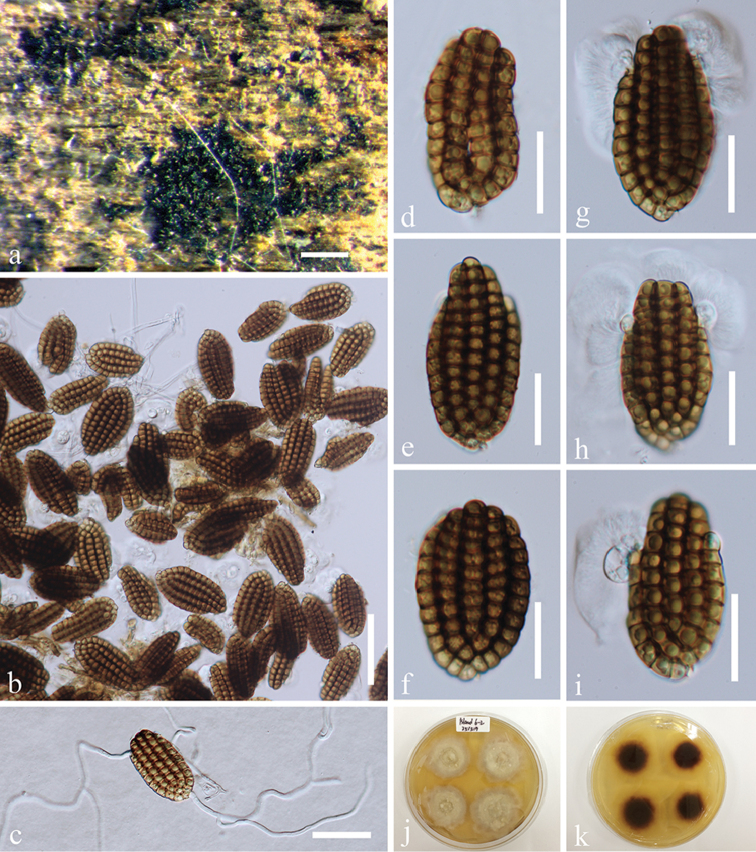
*Dictyosporium tratense* (MFLU 18-1042, holotype). **a** Colonies on submerged wood **b** Squash mount of a sporodochium **c** Germinated conidium **d–i** Conidia **j, k** Culture **j** from above **k** from reverse. Scale bars: **a** = 200 μm, **b** = 50 μm, **c** = 30 μm, **d–i** = 20 μm.

#### Dictyosporium

Taxon classificationFungiPleosporalesDictyosporiaceae

sp.

Figure 7

##### Material examined.

THAILAND. Prachuap Khiri Khan Province, near 12°30.19'N, 99°31.35'E, on decaying wood submerged in a freshwater stream, 25 December 2014, J. van Strien, Site 5-5-1 (MFLU 15-1164), living culture MFLUCC 15-0629 (Additional SSU sequence GenBank MH381760).

##### Notes.

Phylogenetic analyses indicated the isolate *Dictyosporium* sp. (MFLUCC 15-0629) was placed as sister taxon to *D. digitatum* (KH 401), *D. aquaticum* (MF 1318) and *D. stellatum* (CCFC 241241) with good support. The strain *D. digitatum* (KH 401), *D. aquaticum* (MF 1318) and our strain MFLUCC 15-0629, showed the same nucleotide (490 bp) between them for ITS gene regions, while there is only one nucleotide difference between our strain and *D. stellatum* (CCFC 241241). However, the strain *Dictyosporium* sp. (MFLUCC 15-0629) showed seven nucleotides different from *D. digitatum* (yone 280) for ITS gene regions. Morphologically, *D. digitatum* and *D. aquaticum* share the character in having appendages borne at the terminal cells of each conidial arm (Chen et al. 1991, Liu et al. 2015). *Dictyosporium stellatum* differs from *D. digitatum* and *D. aquaticum* in lacking conidial appendages (Crous et al. 2011). In this case, it is difficult to identify our collection based on the recommendations advocated by Jeewon and Hyde (2016) for differentiating species or establishing new species. Thus, we recommend designating this collection as unknown species until enough evidence is available for its identification.

**Figure 7.**
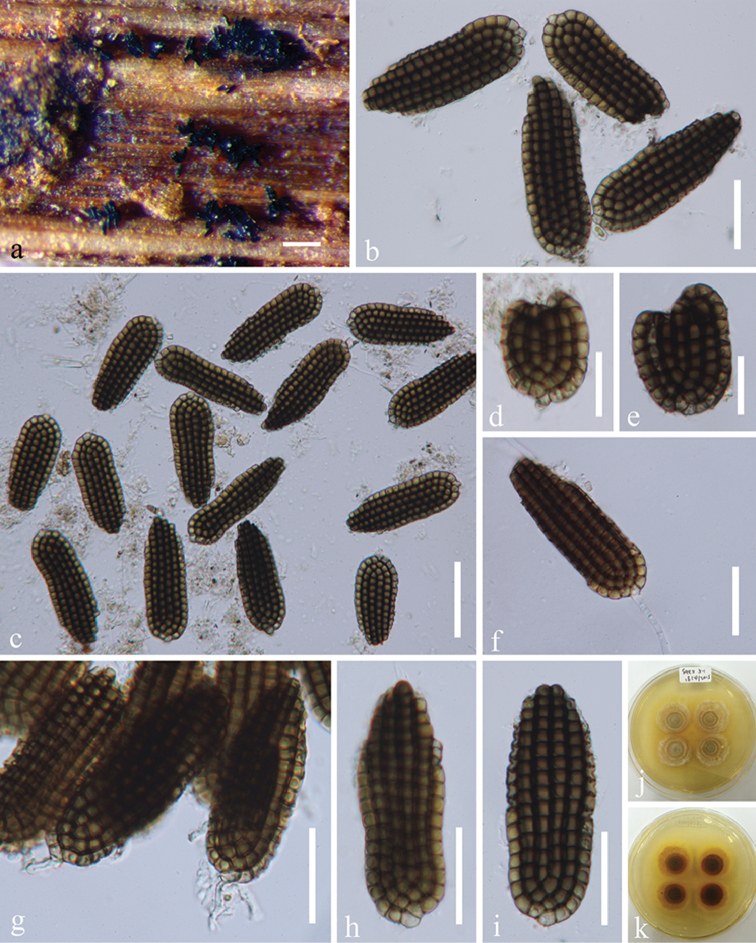
*Dictyosporium* sp. (MFLU 15-1164). **a** Colonies on submerged wood **b** Squash mount of a sporodochium; **c** Germinated conidium **b–e, h, i** Conidia **f** Germinated conidium **g** Conidia with conidiophores **j, k** Culture, j from above, k from reverse. Scale bars: **a** = 200 μm, **b, f–i** = 30 μm, **c** = 50 μm **d, e** = 20 μm.

#### Dictyosporium nigroapice

Taxon classificationFungiPleosporalesDictyosporiaceae

Goh, W.H. Ho & K.D. Hyde, Fungal Diversity 2: 83 (1999)

Figure 8

##### Material examined.

THAILAND. Trat Province, Amphoe Ko Chang, 12°08'N, 102°38'E, on decaying wood submerged in a freshwater stream, 27 April 2017, Y.Z. Lu, YJT 7-1 (MFLU 18-1043, HKAS 102134), living culture MFLUCC 17-2053 (Additional SSU sequence GenBank MH381762).

##### Notes.

Conidia in *Dictyosporium nigroapice* are characterised by conspicuously darker apical cells of the two inner arms, rarely darker at the apex of the outer arms. Morphological characters of this collection well agree with the original diagnosis of the holotype of *D. nigroapice* (Goh et al. 1999).

**Figure 8.**
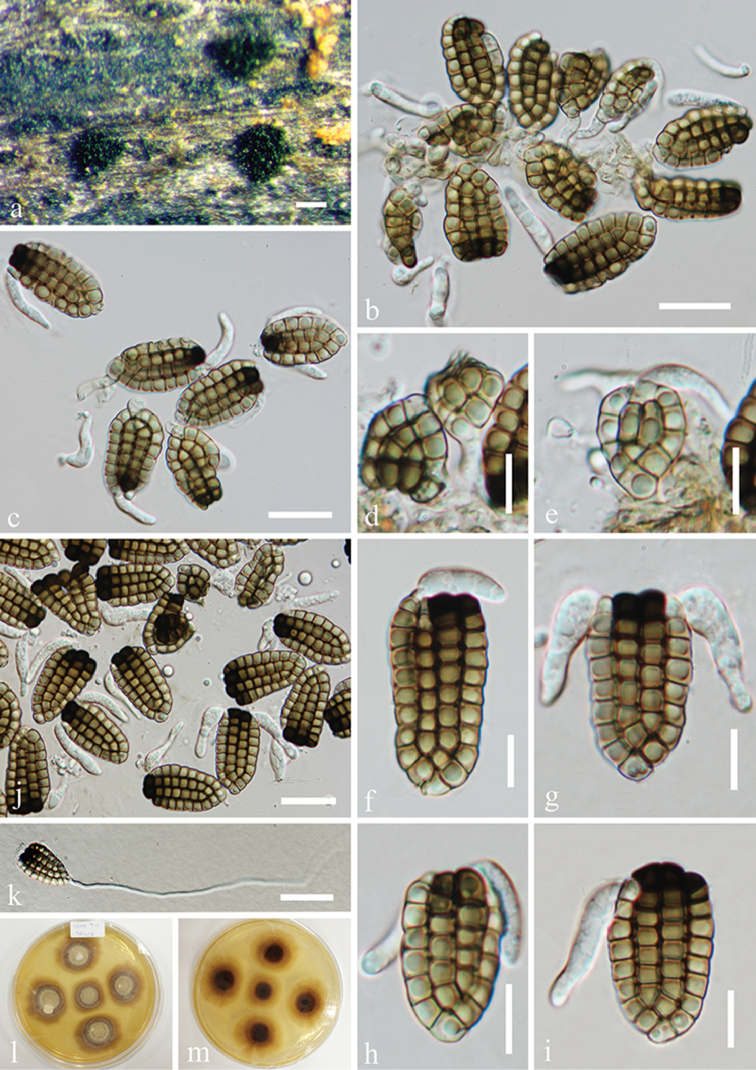
*Dictyosporium nigroapice* (MFLU18-1043). **a** Colonies on submerged wood **b, c** Conidia and conidiophores **d–j** Conidia **k** Germinated conidium **l, m** Culture, l from above, m from reverse. Scale bars: **a** = 100 μm, **b, c, j** = 20 μm, **d–i** = 10 μm, **k** = 30 μm.

## Discussion

Dictyosporiaceae accommodates a holomorphic group of Dothideomycetes, including 12 genera with nine being dictyosporous (Wijayawardene et al. 2017b, Wijayawardene et al. 2018). *Dictyocheirospora* and *Dictyosporium* are the two largest genera in the family. *Dictyosporium* has cheiroid, digitate and complanate conidia without separating arms, while *Dictyocheirospora* is characterised by non-complanate conidia with arms arising from the basal cell and closely gathered at the apex and compact. Thus, *Dictyosporium hydei*, *D. indicum*, *D. musae* and *D. tetraploides* are transferred to *Dictyocheirospora* based on the clear morphological characters. Phylogenetic analyses revealed the placement of *Dictyocheirospora indica* (MFLUCC 15-0056 reference specimen) within *Dictyocheirospora*. We believe that the other three species belong to *Dictyocheirospora* in having similar conidia and appendages to *Dictyocheirospora indica*, although molecular data are unavailable for them.

## Supplementary Material

XML Treatment for Dictyocheirospora bannica

XML Treatment for Dictyocheirospora hydei

XML Treatment for Dictyocheirospora indica

XML Treatment for Dictyocheirospora musae

XML Treatment for Dictyocheirospora rotunda

XML Treatment for Dictyocheirospora tetraploides

XML Treatment for Dictyosporium tubulatum

XML Treatment for Dictyosporium tratense

XML Treatment for Dictyosporium

XML Treatment for Dictyosporium nigroapice

## References

[B1] Abdel-Aziz FA (2016) Two new cheirosporous asexual taxa (Dictyosporiaceae, Pleosporales, Dothideomycetes) from freshwater habitats in Egypt. Mycosphere 7(4): 448–457. 10.5943/mycosphere/7/4/5

[B2] Alves-Barbosa M Costa PMO Malosso E Castañeda-Ruiz RF (2017) Two new species of *Dictyosporium* and *Helminthosporium* (Ascomycota) from the Brazilian Atlantic Forest. Nova Hedwigia 105: 65–73. 10.1127/nova_hedwigia/2017/0401

[B3] Ariyawansa HA Hawksworth DL Hyde KD Jones EBG et al. (2014) Epitypification and neotypification: guidelines with appropriate and inappropriate examples. Fungal Diversity 69: 57–91. 10.1007/s13225-014-0315-4

[B4] Boonmee S D’souza MJ Luo ZL Pinruan U Tanaka K Su H Bhat DJ McKenzie EHC Jones EBG Taylor JE Phillips AJL Hirayama K Eungwanichayapant PD Hyde KD (2016) Dictyosporiaceae fam. nov. Fungal Diversity 80: 457–482. 10.1007/s13225-016-0363-z

[B5] Cai L Zhang KQ McKenzie EHC Hyde KD (2003) New species of *Dictyosporium* and *Digitodesmium* from submerged wood in Yunnan, China. Sydowia 55: 129–135.

[B6] Chen JL Hwang CH Tzean SS (1991) *Dictyosporium digitatum*, a new hyphomycete from Taiwan. Mycological Research 95: 1145–1149. 10.1016/S0953-7562(09)80565-0

[B7] Chomnunti P Hongsanan S Aguirre-Hudson B Tian Q Peršoh D Dhami MK Alias AS Xu J Liu X Stadler M Hyde KD (2014) The sooty moulds. Fungal Diversity 66: 1–36. 10.1007/s13225-014-0278-5

[B8] Corda AC (1836) Mykologische Beobachtungen. Weitenweber’s Beitrage zur gesammtem Natur-und Heilwissenschaften Prague.

[B9] Crous PW Groenewald JZ Shivas RG Edwards J et al. (2011) Fungal Planet Description Sheets: 69–91. Persoonia 26: 108–156. 10.3767/003158511x581723 22025808 PMC3160798

[B10] da Silva SS Castañeda-Ruiz RF Gusmão LFP (2016) New species and records of *Dictyosporium* on *Araucaria angustifolia* (Brazilian pine) from Brazil. Nova Hedwigia 102: 523–530. 10.1127/nova_hedwigia/2015/0325

[B11] Goh TK Hyde KD Ho WH (1999) A revision of the genus *Dictyosporium*, with descriptions of three new species. Fungal Diversity 2: 65–100.

[B12] Hillis DM Bull JJ (1993) An empirical test of bootstrapping as a method for assessing confidence in phylogenetic analysis. Systematic Biology 42(2): 182. 10.2307/2992540

[B13] Ho WH Yanna Hyde KD Hodgkiss IJ (2002) Seasonality and sequential occurrence of fungi on wood submerged in Tai Po Kau Forest Stream, Hong Kong. Fungal Diversity 10: 21–43.

[B14] Huelsenbeck JP Ronquist F (2001) MRBAYES: Bayesian inference of phylogenetic trees. Bioinformatics 17(8): 754–755. 10.1093/bioinformatics/17.8.754 11524383

[B15] Hyde KD Fryar S Tian Q Bahkali AH Xu JC (2016) Lignicolous freshwater fungi along a north-south latitudinal gradient in the Asian/Australian region; can we predict the impact of global warming on biodiversity and function? Fungal Ecology 19: 190–200. 10.1016/j.funeco.2015.07.002

[B16] Hyde KD Goh TK (1998) Fungi on submerged wood in Lake Barrine, north Queensland, Australia. Mycological Research 102: 739–749. 10.1017/s0953756297005868

[B17] Hyde KD Norphanphoun C Abreu VP Bazzicalupo A et al. (2017) Fungal diversity notes 603–708: taxonomic and phylogenetic notes on genera and species. Fungal Diversity 87: 1–235. 10.1007/s13225-017-0391-3

[B18] Index Fungorum (2018) http://www.indexfungorum.org

[B19] Jayasiri SC Hyde KD Ariyawansa HA Bhat DJ et al. (2015) The faces of fungi database: fungal names linked with morphology, phylogeny and human impacts. Fungal Diversity 74(1): 3–18. 10.1007/s13225-015-0351-8

[B20] Jeewon R Hyde KD (2016) Establishing species boundaries and new taxa among fungi: recommendations to resolve taxonomic ambiguities. Mycosphere 7(11): 1669–1677. 10.5943/mycosphere/7/11/4

[B21] Katoh K Standley DM (2013) Multiple sequence alignment software version 7: improvements in performance and usability. Molecular Biology and Evolution 30: 772–780. 10.1093/molbev/mst010 23329690 PMC3603318

[B22] Larget B Simon DL (1999) Markov Chain Monte Carlo algorithms for the Bayesian analysis of phylogenetic trees. Molecular Biology and Evolution 16: 750–759. 10.1093/oxfordjournals.molbev.a026160

[B23] Li WL Luo ZL Liu JK Bhat DJ Bao DF Su HY Hyde KD (2017) Lignicolous freshwater fungi from China I: *Aquadictyospora lignicola* gen. et sp. nov. and new record of *Pseudodictyosporium wauense* from northwestern Yunnan Province. Mycosphere 8(10): 1587–1597. 10.5943/mycosphere/8/10/1

[B24] Liu JK Hyde KD Jones EBG Ariyawansa HA et al. (2015) Fungal diversity notes 1–110: taxonomic and phylogenetic contributions to fungal species. Fungal Diversity 72: 1−197. 10.1007/s13225-015-0324-y

[B25] Miller MA Pfeiffer W Schwartz T (2010) Creating the CIPRES Science Gateway for inference of large phylogenetic trees. Proceedings of the Gateway Computing Environments Workshop (GCE), 14 Nov 2010. New Orleans, LA, 1–8. 10.1109/GCE.2010.5676129

[B26] Nylander J (2008) MrModeltest2 v. 2.3 (Program for selecting DNA substitution models using PAUP*). Evolutionary Biology Centre, Uppsala, Sweden.

[B27] Photita W Lumyong P McKenzie EHC Hyde KD Lumyong S (2002) A new *Dictyosporium* species from *Musa acuminata* in Thailand. Mycotaxon 82: 415–419.

[B28] Pinnoi A Lumyong S Hyde KD Jones EBG (2006) Biodiversity of fungi on the palm *Eleiodoxa conferta* in Sirindhorn peat swamp forest, Narathiwat, Thailand. Fungal Diversity 22: 205–218.

[B29] Pinruan U Hyde KD Lumyong S McKenzie EHC Jones EBG (2007) Occurrence of fungi on tissues of the peat swamp palm *Licuala longicalycata*. Fungal Diversity 25: 157–173.

[B30] Prasher IB Verma RK (2015) Two new species of *Dictyosporium* from India. Phytotaxa 204: 193–202. 10.11646/phytotaxa.204.3.2

[B31] Rannala B Yang Z (1996) Probability distribution of molecular evolutionary trees: a new method of phylogenetic inference. Journal of Molecular Evolution 43: 304–311. 10.1007/pl00006090 8703097

[B32] Rehner S (2001) Primers for Elongation Factor 1-α (EF1-α). http://ocid.NACSE.ORG/research/deephyphae/EF1primer.pdf

[B33] Silva CR Gusmão LFP Castañeda-Ruiz RF (2015) *Dictyosporium amoenum* sp. nov from Chapada, Diamantina, Bahia, Brazil. Mycotaxon 130: 1125–1133. 10.5248/130.1125

[B34] Stamatakis A (2006) RAxML-VI-HPC: maximum likelihood-based phylogenetic analyses with thousands of taxa and mixed models. Bioinformatics 22: 2688–2690. 10.1093/bioinformatics/btl446 16928733

[B35] Stamatakis A Hoover P Rougemont J (2008) A rapid bootstrap algorithm for the RAxML web servers. Syst Biol 57: 758–771. 10.1080/10635150802429642 18853362

[B36] Swofford DL (2003) PAUP*: Phylogenetic analysis using parsimony (*and other methods). Version 4. Sinauer, Sunderland

[B37] Tanaka K Hirayama K Yonezawa H Sato G Toriyabe A Kudo H Hashimoto A Matsumura M Harada Y Kurihara Y Shirouzu T Hosoya T (2015) Revision of the Massarineae (Pleosporales, Dothideomycetes). Studies in Mycology 82: 75–136. 10.1016/j.simyco.2015.10.002 26955201 PMC4774272

[B38] Tsui CKM Berbee ML Jeewon R Hyde KD (2006) Molecular phylogeny of *Dictyosporium* and allied genera inferred from ribosomal DNA. Fungal Diversity 21: 157–166.

[B39] Vilgalys R Hester M (1990) Rapid genetic identification and mapping of enzymatically amplified ribosomal DNA from several *Cryptococcus* species. Journal of Bacteriology 172(8): 4238–4246. 10.1128/jb.172.8.4238-4246.1990 2376561 PMC213247

[B40] Wang RX Luo ZL Hyde KD Bhat DJ Su XJ Su HY (2016) New species and records of *Dictyocheirospora* from submerged wood in north-western Yunnan, China. Mycosphere 7(9): 1357–1367. 10.5943/mycosphere/7/9/9

[B41] White TJ Bruns T Lee SJ Taylor JW (1990) Amplification and direct sequencing of fungal ribosomal RNA genes for phylogenetics. PCR protocols: a guide to methods and applications 18(1): 315–322. 10.1016/B978-0-12-372180-8.50042-1

[B42] Whitton SR McKenzie EHC Hyde KD (2012) Anamorphic fungi associated with Pandanaceae. Springer Netherlands 21: 125–353. 10.1007/978-94-007-4447-9_4

[B43] Wijayawardene NN Hyde KD Lumbsch HT Liu JK et al. (2018) Outline of Ascomycota: 2017. Fungal Diversity 88: 167–263. 10.1007/s13225-018-0394-8

[B44] Wijayawardene NN Hyde KD Rajeshkumar KC et al. (2017a) Notes for genera: Ascomycota. Fungal Diversity 86(1): 1–594. 10.1007/s13225-017-0386-0

[B45] Wijayawardene NN Hyde KD Tibpromma S Wanasinghe DN Thambugala KM Tian Q Wang Y (2017b) Towards incorporating asexual fungi in a natural classification: checklist and notes 2012–2016. Mycosphere 8(9): 1457–1555. 10.5943/mycosphere/8/9/10

[B46] Zhang Y Cai CS Zhao GZ (2017) *Dictyosporium wuyiense* sp. nov. from Wuyi Mountain China. Phytotaxa 314(2): 251–258. 10.11646/phytotaxa.314.2.6

[B47] Zhaxybayeva O Gogarten JP (2002) Bootstrap, Bayesian probability and maximum likelihood mapping: exploring new tools for comparative genome analyses. BMC Genomics 3: 4. 10.1186/1471-2164-3-4 PMC100357 11918828

